# Temporal validation of the electronic health record cancer-associated thrombosis model in patients with low bleeding risk

**DOI:** 10.1016/j.rpth.2026.106830

**Published:** 2026-07-14

**Authors:** Jennifer La, Omid Jafari, Kaelyn Nannini, Nhan V. Do, Mary T. Brophy, Nathanael R. Fillmore, Ang Li

**Affiliations:** 1 Massachusetts Veterans Epidemiology Research and Information Center, Veterans Affairs Boston Healthcare System, Boston, Massachusetts, USA; 2 Section of Hematology & Medical Oncology, Department of Medicine, Boston University School of Medicine, Boston, Massachusetts, USA; 3 Veterans Affairs Boston Healthcare System, Department of Medicine, Harvard Medical School, Boston, Massachusetts, USA; 4 Section of Hematology-Oncology, Department of Medicine, Baylor College of Medicine, Houston, Texas, USA; 5 Section of General Internal Medicine, Department of Medicine, Boston University School of Medicine, Boston, Massachusetts, USA; 6 Department of Medical Oncology, Dana-Farber Cancer Institute, Boston, Massachusetts, USA

**Keywords:** neoplasms, prediction algorithms, validation study, venous thrombosis, thromboemblism

## Abstract

**Background:**

The electronic health record cancer-associated thrombosis (EHR-CAT) model was derived to predict cancer-associated venous thromboembolism (VTE). Prior validations often relied on cancer registry data with reporting delays.

**Objectives:**

We aimed to perform an external temporal validation of the EHR-CAT model among patients at low risk of bleeding using contemporary Veterans Affairs electronic health record data.

**Methods:**

We conducted a retrospective cohort study in patients with active cancer who received systemic therapy within the national Veterans Affairs (VA) health care system from 2017 to 2024. The key inclusion criteria included: (1) having readily extractable data for EHR-CAT calculation without cancer registry data, (2) index date at first or subsequent lines of therapy, (3) exclusion of adjuvant endocrine monotherapy with low risk for VTE, and (4) application of anticoagulant clinical trial exclusion criteria to minimize bleeding risk. Patients from previously published studies were excluded. Systemic therapies were defined using the VA Corporate Data Warehouse, and one line was randomly selected. VTE outcomes were determined using validated VTE–Bidirectional Encoder Representations from Transformers natural language processing from clinical notes. Time-dependent C-statistic and calibration slope were assessed.

**Results:**

Among 65,341 patients with cancer receiving systemic therapy (42,865 first-line and 22,476 second-line or higher), median age was 71.9 years, and 95% were male. Most common cancers included prostate (16.1%), lung (14.8%), lymphoma (11.5%), and bladder (11.1%). The VTE incidence at 6 months was 5.4%. VTE was observed in 1.9%, 4.0%, 4.7%, 6.5%, 9.6%, and 12.3% of patients with EHR-CAT scores 0−, 1, 2, 3, 4, and 5+, respectively (C-statistic 0.67). Calibration slope was 0.876.

**Conclusion:**

The EHR-CAT model, when applied across different lines of therapy, demonstrated similar performance as in previous studies.

## Essentials

• The VTE and bleeding are serious complications for patients with cancer.
• We validated the EHR-CAT risk score in 65,341 veterans with cancer receiving systemic therapy.
• The EHR-CAT risk scores stratified patients into distinct risk categories for VTE by 6 months.
• The risk score had similar performance as original study even after exclusion for bleeding risk.

## 1 introduction

Venous thromboembolism (VTE) is a serious complication in cancer patients, particularly among those undergoing systemic therapy. Cancer increases the risk of thrombotic events by 7-fold, with the highest incidence occurring within the first few months after diagnosis [1]. VTE remains a leading cause of morbidity and mortality in patients with cancer [[2], [3], [4]]. Randomized clinical trials have demonstrated that prophylactic administration of low-molecular-weight heparin or direct oral anticoagulants significantly reduces the relative risk of VTE, with the most substantial absolute risk reductions observed in patients at highest baseline risk [5,6]. Importantly, the increased risk of bleeding associated with prophylactic anticoagulation requires careful patient selection to optimize the net clinical benefit.

Many risk assessment models have been developed to identify cancer patients at elevated risk of VTE [[7], [8], [9], [10]]. The electronic health record (EHR) cancer-associated thrombosis (EHR-CAT) model, a risk assessment model derived from EHR data, has demonstrated superior performance compared with the gold standard Khorana score, nearly doubling the identification of high-risk patients who develop VTE [7]. While the EHR-CAT model has been externally validated in several different cancer populations to have similar performance as in the original study [[11], [12], [13], [14]], other evaluations have noted reduced performance [15,16]. In addition to this performance discrepancy, previous validations of the EHR-CAT model have also been limited largely to patients receiving first-line systemic therapy within 1 year of diagnosis and did not exclude patients with high bleeding risk or those receiving adjuvant endocrine monotherapy (very low VTE risk). Additionally, these studies predominantly utilized cancer registry data, which are subject to reporting delays. To enable future implementation, it is critical to assess the performance of the model performance both in first-line and subsequent lines of systemic therapy using real-time structured data directly from EHRs.

In this study, we leveraged the Veterans Affairs (VA) EHR to externally validate the EHR-CAT model for VTE risk stratification in a retrospective real-world cancer population. To improve outcome ascertainment, we employed a large language model-based natural language processing (NLP) algorithm to identify VTE events [17]. We examined score performance in patients with low bleeding risk based on established clinical trial exclusion criteria. Finally, we excluded any patients previously reported [7], to establish a more contemporary validation cohort.

## 2 Methods

### 2.1 Study design, data source, and participants

We conducted a retrospective cohort study of patients with cancer within the national VA health care system. Using the VA Corporate Data Warehouse, cancer diagnoses were defined using International Classification of Diseases (ICD) codes from January 2017 to June 2024, where ≥2 of the same coding group within 365 days were considered an era. The earliest systemic therapy mapped ±30 days to a cancer ICD code was considered the initiation of systemic therapy, and subsequent lines of therapy were derived from this date.

**Figure 1:**
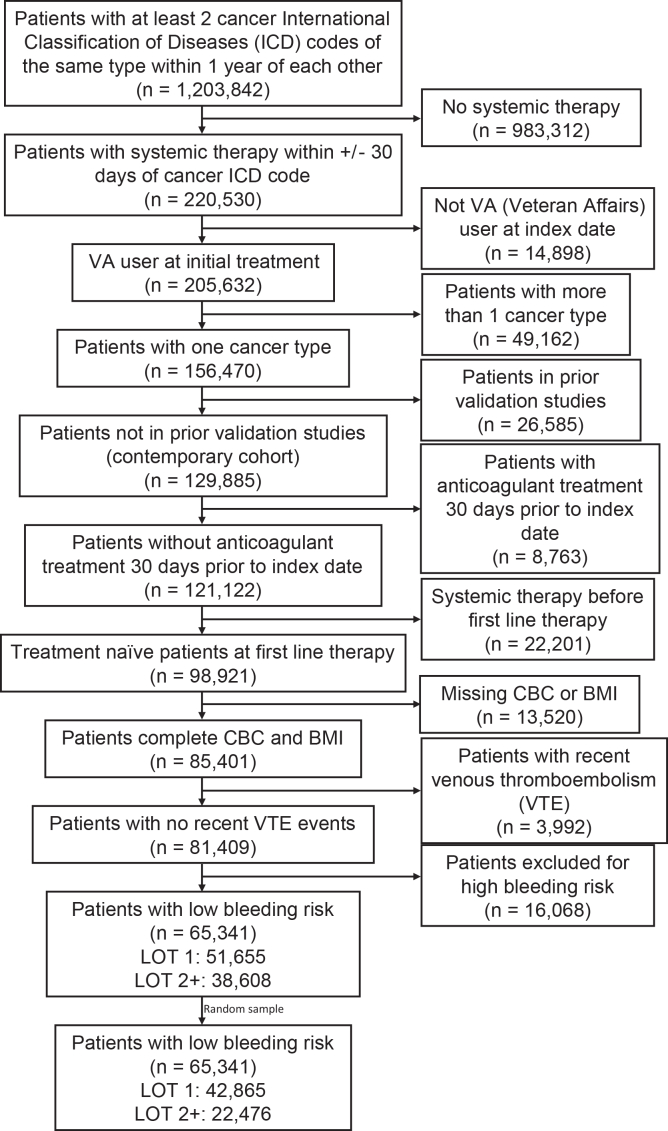
Patient selection flow diagram. BMI, body mass index; CBC, complete blood count.

Exclusion criteria included those without a record of systemic therapy associated with an cancer ICD code, non–primary VA user (≥1 clinical encounter in 2 of the 3 years before index date), presence of multiple primary cancers, inclusion in our previous validation studies, receipt of anticoagulant within 30 days before the index date, documentation of systemic therapy before first-line treatment (treatment not associated with ICD code), missing body mass index (BMI) or complete blood count data, a documented acute VTE event within 6 months before the index date, and high risk for bleeding (see below) (Figure 1). Since BMI and complete blood count was routinely measured for most VA patients at the start of systemic therapy, their absence was associated with infrequent VA encounters and low-quality data, and as such, we avoided performing imputations.

### 2.2 Line of therapy definition and selection

Lines of therapy were defined beginning from the first systemic cancer treatment date. The following common adjuvant endocrine therapies were not considered: anastrozole, bicalutamide, exemestane, goserelin, letrozole, leuprolide, and tamoxifen. All treatments administered within a rolling window of 30 days at the time of initiation date were categorized as the same line. A subsequent line was defined only when a new antineoplastic agent was initiated outside the 30-day window, ensuring that standard cycle lengths of 21 to 28 days were appropriately captured. A new line was also assigned when there was a treatment gap >90 days. This algorithm was applied iteratively to define multiple lines of therapy per patient. One line was randomly selected as the index date for analysis.

### 2.3 EHR-CAT score and bleeding risk covariates

The EHR-CAT score comprises 11 predictors of VTE risk, encompassing reclassified components of the Khorana score alongside cancer-specific and patient-specific factors [7]. Specifically, the risk score included cancer type, cancer stage, treatment type, BMI, white blood cell count, hemoglobin, platelet count, VTE history, recent hospitalization, paralysis or immobility, and Asian race. Each of the 11 predictors provided a numeric score that can be summed together as a final integer score, with caps at 0− and 5+.

Bleeding risk was defined using criteria adapted from clinical trial exclusion parameters and included: weight <40 kg, recent bleeding ICD codes (≤ 90 days on/before treatment date), primary brain cancer or metastasis to the brain, acute leukemia, platelet count < 50 × 10^9^/L, alanine aminotransferase > 156 U/L, bilirubin > 2.4 mg/dL, glomerular filtration rate < 30 mL/min/1.73 m^2^ (≤ 90 days on/before treatment date), prescription of anticoagulants or non-aspirin antiplatelets, or CYP3A-interacting medications (apalutamide, enzalutamide, mitotane, encafenib, adagrasib, tucatinib, rifampin, rifampicin, erythromycin, ketoconazole, levoketoconazole, posaconazole, itraconazole, isavuconazole, ritonavir, cobicistat, nirmatrelvirritonavir, verapamil, dronedarone, carbamazepine, phenytoin, fosphenytoin, phenobarbital, primidone, mifepristone, St. John’s wort ≤ 180 days on/before treatment date).

### 2.4 Outcome definition

The primary outcome was VTE at 6 months after treatment. VTE was defined as pulmonary embolism and/or lower/upper extremity deep vein thrombosis and was ascertained using the VTE–Bidirectional Encoder Representations from Transformers NLP model using clinical notes (85% precision, 92% recall) [17]. Patients were followed from the treatment until the first occurrence of VTE, death, loss to follow-up (gap of ≥ 90 days without clinical encounters), or administrative censoring on September 28, 2025.

### 2.5 Statistical analysis

To evaluate predictive performance, the EHR-CAT score was calculated for each patient. Participants were categorized based on their numeric risk score using previously established cutoffs [7,13]. The 6-month cumulative incidences were calculated using a cumulative incidence function with death as a competing event. Similar to the initial studies, discrimination was assessed using the time-dependent C-statistic at 6 months to account for interval censoring (R package *survivalROC*) [18]. Calibration was evaluated by plotting predicted versus observed VTE risk across numeric score strata. Since the model is a simple additive score, the predicted risk was based on the estimated cumulative incidence from the initial derivation study [7].

## 3 Results and Discussion

The final analytic cohort included 65,341 patients with cancer who initiated a new line of systemic therapy (42,216 first-line and 22,476 second-line or higher) between January 2017 and June 2024. Notably, 16,068 patients (19.7%) were excluded for potential risk of bleeding based on anticoagulation clinical trial exclusion criteria. The median age was 71.93 (IQR, 66.00-76.51) years, and 95.0% were male. The racial and ethnic breakdown included: White (71.0%), Black (19.2%), Hispanic (5.0%), and Asian (1.5%). Most common cancer types included prostate (16.1%), lung (14.8%), and lymphoma (11.5%). A substantial portion of the patients were diagnosed with stage III (27.7%) and IV disease (34.2%). In terms of the original Khorana score risk components, 11.1% of patients had hemoglobin < 10 g/dL, 14.6% had white blood cell count > 11 cells/μL, 10.6% had platelet count ≥ 350 cells/μL, and 11.1% had BMI ≥ 35 kg/m^2^. Additional components of the EHR-CAT showed that 15.8% had recent hospitalization, 1.2% had paralysis, 5.2% had a history of VTE, and 28.8% had targeted or endocrine therapy at the index date (Table). The cohort included 14,203 (21.7%), 11,801 (18.1%), 14,734 (22.5%), 12,394 (19.0%), 7406 (11.3%), and 4803 (7.3%) patients with EHR-CAT scores of 0, 1, 2, 3, 4, and 5+, respectively.

**Table:** Baseline characteristics in patients with cancer after random sampling by line of therapy and exclusion by bleeding risk.

Characteristic	Value
*N*	65,341
Age, median **(**IQR**)**	71.93 (66.00, 76.51)
Male gender, *n* (%)	62,051 (95.0)
Race and ethnicity, *n* (%)	
Non-Hispanic White	46,413 (71.0)
Non-Hispanic Black	12,516 (19.2)
Hispanic	3248 (5.0)
Non-Hispanic Asian Pacific Islander/Alaskan Natives/American Indian	1007 (1.5)
Unknown	2157 (3.3)
BMI, kg/m^2^, median **(**IQR**)**	27.25 (23.85, 31.19)
Cancer type, *n* (%)	
Prostate	10,528 (16.1)
Lung	9674 (14.8)
Lymphoma	7516 (11.5)
Bladder	7222 (11.1)
Gastric and esophageal	5601 (8.6)
Head and neck	4408 (6.7)
Liver	4042 (6.2)
Multiple myeloma	2980 (4.6)
Other solid tumor	2824 (4.3)
Chronic leukemia	2697 (4.1)
Kidney	2248 (3.4)
Melanoma	1937 (3.0)
Pancreas	1688 (2.6)
Breast	1290 (2.0)
Biliary and gallbladder	452 (0.7)
Testicular	234 (0.4)
Type of therapy, *n* (%)	
Cytotoxic chemotherapy	40,216 (61.5)
Immune checkpoint inhibitor	9583 (14.7)
Targeted therapy	18,369 (28.1)
Advanced endocrine therapy	6285 (9.6)
Line of therapy, *n* (%)	
First	42,865 (65.6)
Second+	22,476 (34.4)
Relevant laboratory result^a^, median IQR^a^	
WBC, cells/μL	7.15 (5.56, 9.27)
Hemoglobin, g/dL	13.00 (11.40, 14.30)
Platelet, 10^3^/μL	222.00 (173.00, 281.00)
Alanine aminotransferase, units/L	19.00 (14.00, 28.00)
Creatinine, mg/dL	1.00 (0.85, 1.25)
Total bilirubin, mg/dL	0.56 (0.40, 0.80)
Original Khorana score risk components, *n* (%)	
WBC >11 (+1)	9567 (14.6)
Hemoglobin <10 (+1)	7241 (11.1)
Platelet ≥350 (+1)	6951 (10.6)
BMI ≥35 (+1)	7229 (11.1)
Additional her-CAT components, *n* (%)	
Cancer type risk^b^	
Low (0)	33,372 (51.1)
Intermediate (+1)	3410 (5.2)
High (+2)	24,228 (37.1)
Very high (+3)	4331 (6.6)
Advanced cancer stage (III-IV) (+1)	40,469 (61.9)
Recent hospitalization (+1)	10,329 (15.8)
History of paralysis (+1)	759 (1.2)
History of VTE (remote) (+1)	3387 (5.2)
Targeted or endocrine therapy (−1)	18,841 (28.8)
Asian race (−1)	1007 (1.5)
Composite EHR-CAT score, *n* (%)	
0	14,203 (21.7)
1	11,801 (18.1)
2	14,734 (22.5)
3	12,394 (19.0)
4	7406 (11.3)
5	4803 (7.4)

BMI, body mass index; EHR-CAT, electronic health record-cancer-associated thrombosis; VTE, venous thromboembolism; WBC, white blood cell.

a The laboratory results shown here reflect values after exclusion for bleeding risk: weight <40 kg, recent bleeding event, brain cancer or metastasis, acute leukemia, platelet count <50 × 10^9^/L, alanine aminotransferase >156 U/L, bilirubin >2.4 mg/dL, glomerular filtration rate <30 mL/min/1.73 m^2^, prescription of anticoagulants or non-aspirin antiplatelets, or CYP3A-interacting medications.

b Very high (+3): pancreas, stomach, esophageal, biliary/gallbladder; high (+2): lung, bladder, testicular, kidney, ovarian, uterine, brain, soft tissue sarcoma (exclude gastrointestinal stromal tumor), multiple myeloma, diffuse large B cell lymphoma, primary central nervous system lymphoma, Burkitt’s lymphoma, lymphoblastic lymphoma, systemic (noncutaneous) T-cell lymphoma; intermediate (+1): colorectal, intestinal; low (+0): all other cancers not previously listed.

**Figure 2:**
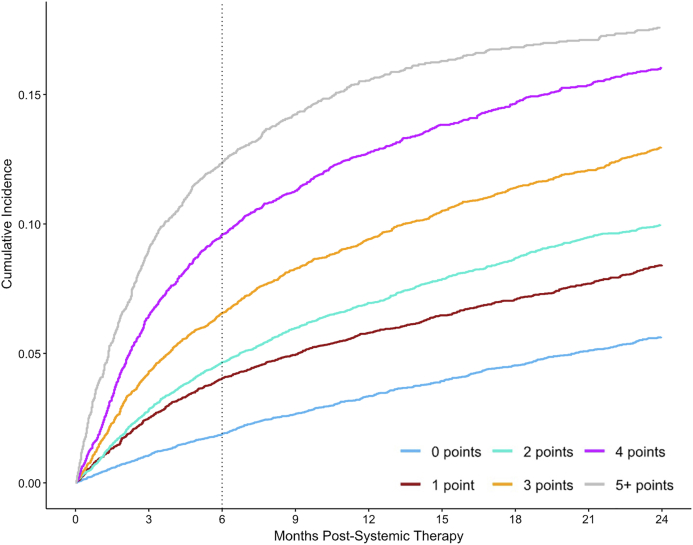
Incidence of venous thromboembolism stratified by the electronic health record-cancer-associated thrombosis (EHR-CAT) model in patients with low bleeding risk.

**Figure 3:**
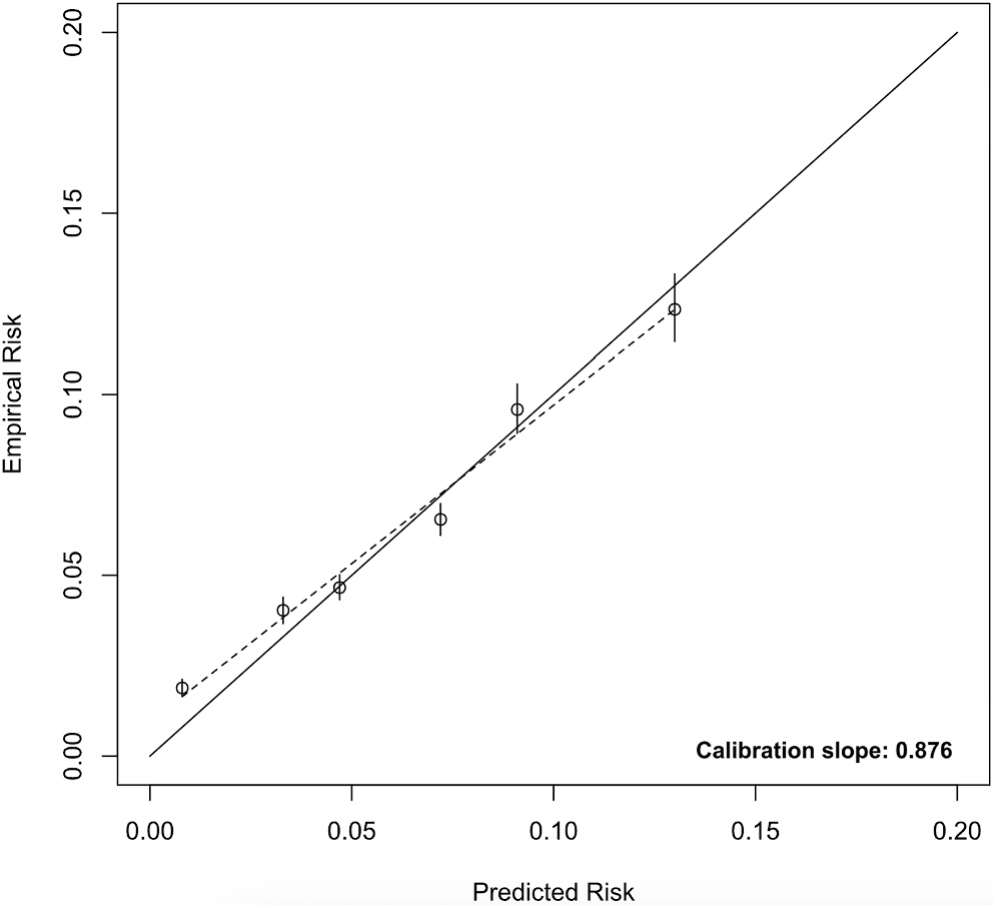
Calibrat ion plot for the EHR-CAT model. Calibration plot and calibration slope are shown here to evaluate the model calibration. The y-axis is the observed probability (current cohort) whereas the x-axis is the predicted probability (original EHR-CAT model). The points represent the score cutoffs from 0− to 5+. The vertical lines represent the bootstrapped confidence interval. EHR-CAT, electronic health record-cancer-associated thrombosis.

The overall incidence of VTE within 6 months was 5.4%. Among the 65,341 patients included, 9467 (14.5%) died within 6 months of the index date. For the overall cohort, the VTE incidence at 6 months was 1.9% (254 of 14,203) for a score of 0, 4.0% (464 of 11,801) for a score of 1, 4.7% (668 of 14,734) for a score of 2, 6.5% (801 of 12,394) for a score of 3, 9.6% (700 of 7406) for a score of 4, and 12.3% (590 of 4803) for a score of 5+ (Figure 2). The model discrimination for the overall cohort had a time-dependent C-statistic of 0.67. The calibration plot had a slope of 0.876, suggesting that the model slightly underestimated the risk in the lowest risk groups (Figure 3).

In this contemporary cohort of patients with cancer, we externally validated the EHR-CAT score using structured EHR data across multiple lines of systemic therapy, rather than relying on cancer registry information or exclusively first-line treatment. While EHR-CAT is not a true dynamic model, the use of dynamic data input at different lines of therapy can likely overcome changes in static variables. We excluded patients deemed to have low VTE risk (those receiving endocrine monotherapy, commonly used for adjuvant prostate or breast cancer) and those deemed high bleeding risk based on exclusion criteria commonly applied in clinical trials evaluating anticoagulant efficacy in cancer populations [5,6]. Our findings demonstrate that the EHR-CAT score exhibited modest discrimination, similar to what was found in our prior study [7], when evaluating VTE risk in a random sample of patients receiving various lines of therapy and can be applied directly using EHR data within the VA population.

There have been other validation studies for the EHR-CAT risk score [[11], [12], [13], [14]]. Several studies that identified eligible patients using cancer registries in the United States and Denmark showed similar performance as the original score (C-statistic 0.68-0.71) [11,13,15,19]. In contrast, a prospective cohort study from the Vienna General Hospital with 806 patients of selective high-risk cancer types with advanced-stage disease showed a decreased performance (C-statistic 0.55) [16]. These differences were likely driven by the type of cancers at enrollment, since cancer types with low risk for VTE, such as early-stage breast and prostate cancer, as well as hematologic malignancies, are underrepresented in prospective cohort studies that focus on advanced solid tumors. In addition to externally validating the risk model, the present study provides several additional methodological advantages, including the direct extraction of risk score element from EHRs, the exclusion of potential bleeding risks, and the inclusion of later lines of therapy, to more accurately represent a real-world population more likely to be considered for prophylactic anticoagulation.

Nonetheless, there are limitations that merit consideration. First, this study was conducted within the VA health care system, which represents a distinct patient population characterized predominantly by older male veterans with a high burden of comorbid illness. Consequently, findings here may not generalize to the broader cancer population, who have significantly lower prevalence of prostate and lung cancers and higher prevalence of breast and ovarian cancers [20]. Furthermore, certain components of the risk model required ICD 10th Revision, Clinical Modification codes that are not readily available outside of the United States; therefore, future work on model simplification is needed for true integration with EHR systems worldwide. Second, as the VA is the largest integrated health care system in the United States offering free or highly subsidized care, its EHR data are likely to be more comprehensive and feature longer follow-up periods than those available in other settings. This may limit the generalizability of our findings to systems with less complete or fragmented data capture. Third, our line of therapy algorithm relies on a rule-based approach that classifies treatment changes using a 30-day rolling window or a 90-day gap to define new lines of therapy. As with any structured-data algorithm, misclassification is possible, particularly in cases where therapy changes occur outside these windows. Furthermore, ongoing adjuvant endocrine therapy such as single-agent leuprolide was not counted as systemic therapy as this was often not administered by oncologists at the VA; therefore, we missed an opportunity to validate the risk model’s performance in this very low-risk group. Fourth, while we accounted for competing risk of death in model calibration, our discrimination metric using *survivalROC* only accounted for time-dependent censoring without the competing risk of death. Fifth, we used an approximation method to identify patients at risk for bleeding by utilizing common clinical trial exclusion criteria, and the accuracy of such approach outside of clinical trials has not been validated. Finally, when patients had multiple lines of therapy, we used random sampling to select one line but excluded anyone with recent acute VTE events before that treatment initiation; this approach increased the precision of VTE capture but likely decreased the actual incidence of VTE in the cohort. Future work on improved NLP algorithm to capture VTE recurrence is needed to fully analyze the performance of the risk score dynamically.

In conclusion, we externally and temporally validated the EHR-CAT model using structured EHR data across various lines of cancer therapy and within patients with low bleeding risk. Collectively, our findings indicate that the EHR-CAT score has consistent performance for veterans in contemporary years.
